# Chinese Herbal Medicine for Wilson's Disease: A Systematic Review and Meta-Analysis

**DOI:** 10.3389/fphar.2019.00277

**Published:** 2019-03-29

**Authors:** Meng-Bei Xu, Pei-Qing Rong, Ting-Yu Jin, Pei-Pei Zhang, Hai-Yong Liang, Guo-Qing Zheng

**Affiliations:** Department of Neurology, The Second Affiliated Hospital and Yuying Children's Hospital of Wenzhou Medical University, Wenzhou, China

**Keywords:** Wilson's disease, Chinese herbal medicine, ATP7B, anti-oxidation, systematic review

## Abstract

Wilson's disease (WD) is a rare autosomal recessive inherited disorder of chronic copper toxicosis. Currently, Chinese herbal medicines (CHM) is widely used for WD. Here, we conducted an updated systematic review to investigate the efficacy and safety of CHM for WD and its possible mechanisms. Randomized-controlled clinical trials (RCTs), which compared CHM with Western conventional medicine or placebo for WD, were searched in six databases from inception to July 2017. The methodological quality was assessed using 7-item criteria from the Cochrane's collaboration tool. All the data were analyzed using Rev-Man 5.3 software. Eighteen studies involving 1,220 patients were identified for the final analyses. A score of study quality ranged from 2/7 to 4/7 points. Meta-analyses showed that CHM could significantly increase 24-h urinary copper excretion and improve liver function and the total clinical efficacy rate for WD compared with control (*p* < 0.05). Additionally, CHM was well tolerated in patients with WD. The underlying mechanisms of CHM for WD are associated with reversing the ATP7B mutants, exerting anti-oxidation, anti-inflammation, and anti-hepatic fibrosis effects. In conclusion, despite the apparent positive results, the present evidence supports, to a limited extent because of the methodological flaws and CHM heterogeneity, that CHM paratherapy can be used for patients with WD but could not be recommended as monotherapy in WD. Further rigorous RCTs focusing on individual CHM formula for WD are warranted.

## Introduction

Wilson's disease (WD) is a rare autosomal recessive inherited disorder that causes copper poisoning in the body, predominantly in the liver and the brain (Walshe, 2009). The global prevalence of WD is between 1 in 5,000 and 1 in 30,000 (Gomes and Dedoussis, 2015). Epidemiological studies have shown a higher incidence and prevalence of WD in China than in western countries (Hu et al., 2011). The WD gene was identified as the trans-membrane copper transporter ATP7B in hepatocytes (Bull et al., 1993; Petrukhin et al., 1993). An absent or reduced function of ATP7B protein causes decreased hepatocellular excretion of copper into bile. In WD, the ever-increasing positive copper balance overwhelms the copper chaperones (copper-binding proteins), causing elevated levels of free copper and copper-induced tissue injury (Patil et al., 2013). Copper metabolism disorder results in multifaceted neurological, hepatic and psychiatric symptoms (Brewer, 2009). When left untreated, WD is fatal. With early diagnosis and appropriate treatment, patients can obtain excellent prognosis (Roberts and Schilsky, 2008; Coffey et al., 2013). Currently, medical treatments and liver transplantation are two main therapeutic approaches that can achieve the generation of a negative copper balance (Hedera, 2017). The EASL Clinical Practice Guidelines of Wilson's disease by European Association for Study of Liver recommended D-penicillamine, trientine, zinc, tetrathiomolybdate, and dimercaprol as medications. However, many side effects such as nephrotoxicity, dermatological toxicity, bone marrow toxicity, severe thrombocytopenia, and total aplasia have been observed in patients with lifelong pharmacological therapy (European Association for Study of Liver, 2012). Liver transplantation is an effective treatment for patients of WD with acute liver failure but it is used only in particular scenarios because of the risks including relatively low engrafting efficiency and lifelong immunosuppression (Filippi and Dhawan, 2014). Thus, an alternative and/or complementary strategy for WD is increasingly sought.

Chinese herbal medicine (CHM) is widely used for WD in the clinic (Ren et al., 1997; Han et al., 1999, 2014; Hong et al., 2000; Cui and Zhao, 2001; Xiao, 2003; Xue et al., 2007; Zhang, 2007; Chen and Wang, 2008, 2010; Wang et al., 2010; Xu et al., 2012a,b; Hu, 2014; Zhang et al., 2014a,b; Fang, 2015; Jiang, 2016; Li et al., 2016), and has been extensively tested by experimental research (Zhang et al., 2011; Lin et al., 2015). Pharmacological studies have shown that CHM can improve the urinary copper excretion and hepatic fibrosis, and protect the brain, liver and kidney (Lutsenko et al., 2007). These beneficial effects are associated with ATP7B gene reversing, anti-oxidant functions, anti-inflammatory actions and suppression of apoptosis (Rosencrantz and Schilsky, 2011). Our group has demonstrated that CHM brings benefits to some patients with WD (Wang et al., 2012). In addition, emerging randomized-controlled clinical trials (RCTs) continuously report the effectiveness and safety of CHM for WD. Therefore, in the present study we aimed to conduct an updated systematic review of CHM for WD focusing on the clinical evidence and possible mechanisms.

## Methods

### Database and Search Strategies

Two trained researchers systematically searched the following databases from their inception to July 2017: PubMed, Cochrane Central Register of Controlled Trials, Chinese National Knowledge Infrastructure, Chinese VIP information and WanFang database. The search strategy of PubMed was as follows, and it was modified to suit other English or Chinese databases.

PubMed search strategy:

#1. Wilson's disease [mh]
#2. Hepatolenticular degeneration [mh]
#3. Copper storage disease [tiab]
#4. Progressive lenticular degeneration [tiab]
#5. or/1-4
#6. Medicine, Chinese Traditional [mh]
#7. Herbal Medicine [mh]
#8. Integrative Medicine [mh]
#9. Traditional Chinese medicine [tiab]#10. herb*[tiab]
#11. or/6-10
#12. #5 and #11
#13. Randomized controlled trial [pt]
#14. Controlled clinical trial [pt]
#15. Randomized [tiab]
#16. placebo [tiab]
#17. drug therapy [sh]
#18. randomly [tub]
#19. groups [tub]
#20. or/13–19
#21. animals [mph] not (humans [min] and animals [min])
#22. 20 not 21
#23. #12 and #22

### Eligibility Criteria

#### Types of Studies

Only RCTs were included, irrespective of population characteristics, blinding, publication status, and language. Quasi-RCTs, such as those in which patients were allocated according to date of birth and order of admission number, were excluded.

#### Types of Participants

We included participants with a diagnosis of WD, according to Chinese Yang Renmin criteria (1995) (Yang, 1995), Chinese Medical Association of Neurology Guidelines for the diagnosis and treatment of hepatolenticular degeneration (2008) (Chinese Medical Association of Neurology, 2008), American Association for the Study of Liver Diseases practice guidelines of Wilson Disease (2008) (Roberts and Schilsky, 2008), and European Association for the Study of the Liver clinical practice guidelines: Wilson's disease (2012) (European Association for Study of Liver, 2012), regardless of age, gender, disease course and severity. The other diagnostic criteria with comparable definitions were also used.

#### Types of Interventions

Analyzed interventions were CHM monotheism or adjunct therapy using any form, any dose or any administrated methods. Comparator treatments were placebo or Western conventional medication (WCM) (Chinese Medical Association of Neurology, 2008; European Association for Study of Liver, 2012). WCM refers to the combination of needed therapies of the following aspects according to the EASL clinical practice guidelines of WD (European Association for Study of Liver, 2012): (1) General supportive care and low copper diet; (2) Medical therapy: D-penicillamine, trientine, zinc, tetrathiomolybdate, or dimercaprol; (3) Liver transplantation. Chinese guideline for diagnoses and treatment of WD (Chinese Medical Association of Neurology, 2008) is similar to the EASL guideline; however, some recommended drugs such as Trientine are not accessible in China, whereby Dimercaprol, including dimercaptosuccinicacid (DMSA) or sodium dimercaptosulphonate (DMPS), are recommended and commonly used for patients with WD. Thus, DMSA or DMPS used as control is also included. Studies comparing one kind of CHM therapy to another CHM were excluded.

#### Types of Outcome Measures

The primary outcome measures were: (1) the amount of copper excreted in the urine in a 24 h period, liver function, and the indicator of hepatic fibrosis; (2) clinical deficit score: the Unified Wilson's Disease Rating Scale (Leinweber et al., 2008) or the Novel Global Assessment Scale (GAS) for Wilson's Disease (Aggarwal et al., 2009); (3) imaging: Brain MRI and functional neuroimaging. The secondary outcome measures were: the total clinical effective rate, laboratory values and adverse events.

#### Selection and Data Extraction

The data were extracted using a standardized data extraction form, including study design, eligibility criteria, characteristics of the sample, the course of treatment, interventions, outcomes, the constituent of CHM and pharmaceutical quality control. Reasons for the exclusion of studies were recorded. Any disagreements were resolved by discussion with or by involving a third author.

### Assessment of Risk of Bias

The RoB of included articles was assessed using the 7-item criteria from the Cochrane' s collaboration tool (Higgins et al., 2011). Two authors independently evaluated the study quality, and the final result was identified by discussion when countering the disagreement.

### CHM Composition

The frequency of use of the particular herb was calculated and those used at a high frequency were described in detail.

### The Reporting Completeness of the Clinical Studies

In order to assess the reporting completeness with a rating system quality of the clinical studies, we used a rating system according to our previous articles (Wang et al., 2019) as follows: (1) high quality: full information about the botanical material is provided, including a voucher specimen; (2) moderate quality: only partial information about the botanical material is provided and a voucher specimen is missing; there are taxonomic inaccuracies; (3) low quality: inadequate information and overall taxonomically is inadequate.

### Statistical Analysis

The pooled analyses were carried out with RevMan 5.3 software. Heterogeneity was assessed using the Cochrane Q-statistic test (*p* < 0.05 was considered statistically significant) and the *I*^2^-statistic test. A fixed effects model (*I*^2^ < 50%) or a random effects model (*I*^2^ > 50%) was used depending on the value of *I*^2^. Funnel plots were used to visually estimate publication bias. We calculated the standard mean difference (SMD) with 95% Confidence Intervals (CIs). Sensitivity analyses omitting everyone, which study at a time from the original analysis were conducted to demonstrate our main results to be robust.

## Results

### Description of Studies

**Figure 1:**
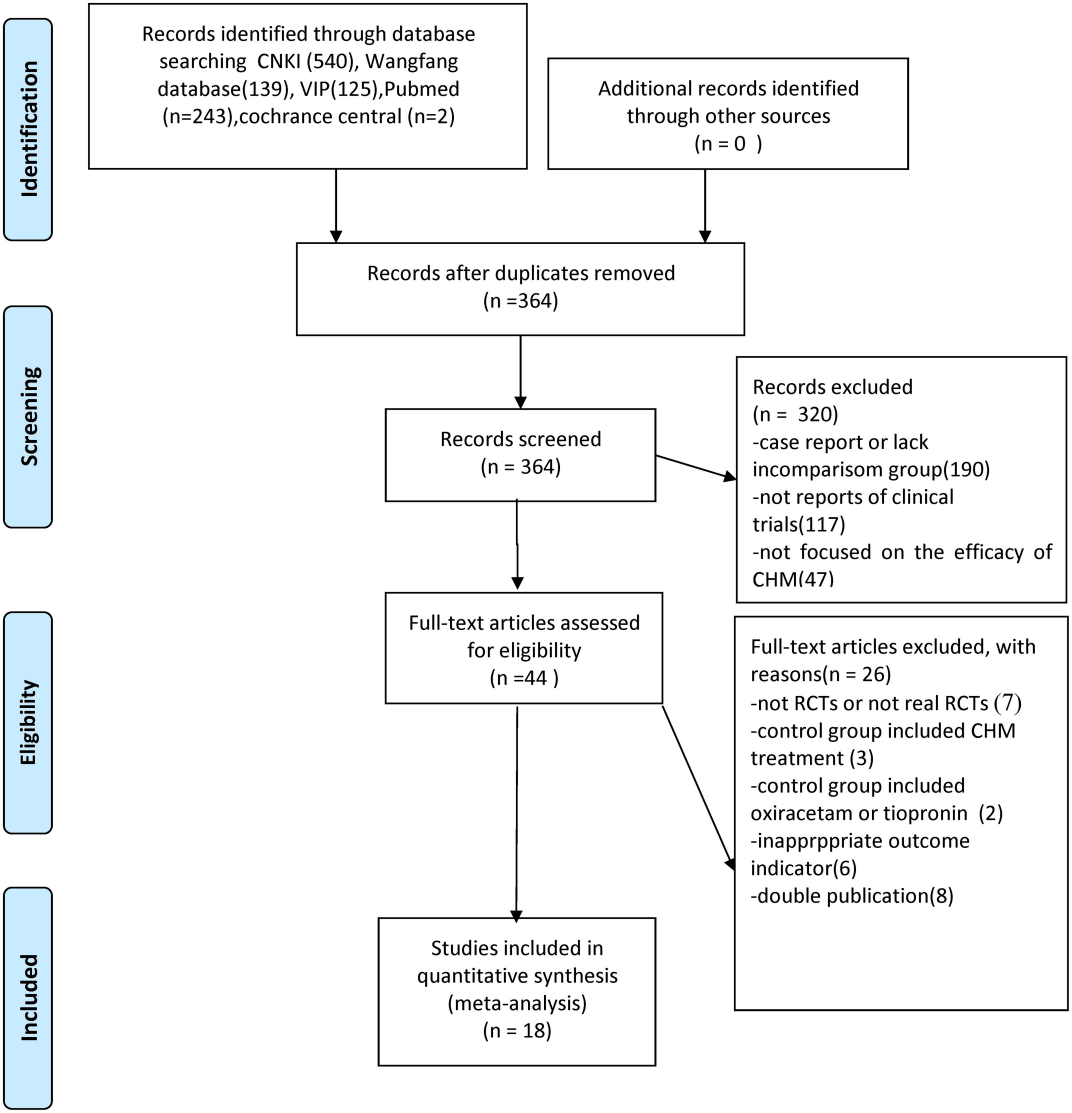
Summary of the process for identifying candidate studies Adapted from Moher et al. (2009).

We identified 1,049 hints, of which 364 articles remained after removal of duplicates. Through screening titles and abstracts, 320 studies were excluded because they were case reports, they lacked a comparison group, they were not CHM studies or reports of clinical trials. After full-text evaluation of the remaining 44 articles, 26 studies were excluded for the following reasons: (1) 7 articles were not RCTs; (2) 3 articles included CHM treatment in control groups; (3) 2 articles included oxiracetam or tiopronin in control group; (4) 6 articles have inappropriate outcome measures; (5) 8 articles were suspected of being published more than once. Eventually, 18 eligible studies were identified (Figure 1).

### Study Characteristics

Eighteen studies with 1,220 participants were included from 1997 to 2016. The sample size ranged from 33 to 146 with an age of 3 to 59 years. The duration of diseases lasted from 1 month to 31 years. The course of treatment ranged from 28 to 90 days. Five studies (Han et al., 2014; Hu, 2014; Zhang et al., 2014a; Fang, 2015; Jiang, 2016) were diagnosed according to Chinese Medical Association of Neurology (2008), 9 studies (Han et al., 1999; Hong et al., 2000; Xue et al., 2007; Zhang, 2007; Chen and Wang, 2010; Wang et al., 2010; Xu et al., 2012a,b; Zhang et al., 2014b) were diagnosed according to Yang criteria (1995) and 4 studies used comparable definitions (Ren et al., 1997; Cui and Zhao, 2001; Xiao, 2003; Chen and Wang, 2008). Three studies (Han et al., 1999; Chen and Wang, 2008, 2010) used CHM monotherapy, and the others used CHM paratherapy. The control group used penicillamine (Xiao, 2003; Zhang, 2007; Chen and Wang, 2008, 2010), DMSA (Ren et al., 1997; Xiao, 2003; Zhang et al., 2014a; Fang, 2015), DMPS (Hong et al., 2000; Xue et al., 2007; Wang et al., 2010; Xu et al., 2012a,b; Han et al., 2014; Hu, 2014; Zhang et al., 2014b; Jiang, 2016), Zinc sulfate (Cui and Zhao, 2001; Xiao, 2003). The characteristics of the 18 trials are summarized in Table 1. In four studies (Han et al., 1999; Hong et al., 2000; Xiao, 2003; Chen and Wang, 2010), the preparations were made in hospitals including the associated pharmaceutical quality control. Six studies (Wang et al., 2010; Xu et al., 2012b; Han et al., 2014; Zhang et al., 2014a; Fang, 2015; Jiang, 2016) used a commercial preparation and in 8 studies (Ren et al., 1997; Cui and Zhao, 2001; Xue et al., 2007; Zhang, 2007; Chen and Wang, 2008; Xu et al., 2012a; Hu, 2014; Zhang et al., 2014b) no data on quality control were reported. The constituent of CHM and pharmaceutical quality control in each included study was listed in detail in Table 2.

**Table 1:** Characteristics of the included studies.

**References**	**Eligibility criteria**	**Study design**	**Interventions(n) drug**		**Sample size**	**Sample and characteristics (male/female), age, duration**		**Course of treatment**	**Course of treatment Outcomes**	**Intergroup differences**
			**Trial**	**Control**		**Trial**	**Control**			
Jiang, 2016	CMAN Standard	RCT	GDL+DMPS	DMPS	60	16/14 25.67 ± 4.82	15/15 25.10 ± 4.63	32 d	1. Vascular injury factor 1.1 Homocysteine 1.2 Von Willebrand Factor 1.3 Thrombomodulin 1.4 Endothelial cell protein C receptor 2. Ultrasound cerebral vessels function 3. Transcranial doppler 4. Perfusion-weighted imaging	1.1.*p* > 0. 051.2 *p* < 0. 011.3. *p* < 0. 051.4 *p* < 0. 012. *p* > 0. 053. *p* < 0. 054. *p* < 0. 05
Fang, 2015	CMAN Standard	RCT	GDL+DMSA	DMSA	60	16/14 21.53 ± 8.35 -	15/15 22.03 ± 9.01 -	30 d	1. Cardiac function 1.1 Electrocardiogram ECG 1.2 Cardiacultrasound:EF 1.3 Myocardial enzyme spectrum (CK,CK-MBL,DH) 2. Blood trace of Ceruloplasmin, Cu2+,copper oxidase 3.24 h excretion of urinary copper 4. Clinical symptoms 5. Adverse effect	1.1 *p* < 0.051.2 *p* < 0.051.3 *p* < 0.052. *p* > 0.053. *p* < 0.05
Zhang et al., 2014a	CMAN Standard	RCT	GDL+DMSA	DMSA	70	20/15 19.36 ± 4.85 10 mo to 20 y	19/16 18.16 ± 4.02 2 m to 18 y	30 d	1.urinary microalbumin	1. *p* < 0.05
Zhang et al., 2014b	YanRenMing Standard	RCT	A:GDT + DMPS B:GDT	C:DMPS	61	38/23a 17.64 ± 6.28 6 m−19y		46 d	1. The indicator of portal circulation PVFV, SVFV 2. 24 h excretion of urinary copper	1. *p* > 0.05 2. *p* < 0.01
Hu, 2014	CMAN Standard	RCT	GDT+DMPS	DMPS	67	16/19 22.66 ± 8.17 5 mo−30 y	15/17 21.97 ± 8.42 3 m−27 y	30 d	1. Pulmonary ventilation function 1.1 FVC % 1.2 FEV1.0/FVC% 2.blood trace of Ceruloplasmin, Cu2+,copper oxidase 3. 24 h excretion of urinary copper 4. adverse effect	1.1 *p* < 0.051.2 *p* > 0.052. *p* > 0.053. *p* < 0.05
Han et al., 2014	CMAN Standard	RCT	GDL+DMPS	DMPS	52	12/19 22.31 ± 4.62 -	11/10 20.63 ± 5.79 -	84 d	1. MMSE 2. MoCA	1. *p* < 0.012. *p* < 0.01
Xu et al., 2012a	YanRenMing Standard	RCT	GDT+DMPS +GSH	DMPS+GSH	56	25/11 21.0 6.6	14/6 22.5 6.3	62 d	1. Clinical symptoms, 2. 24 h excretion of urinary copper 3. Liver function 4. Adverse effect	1. *p* < 0.052. *p* < 0.013. *p* > 0.05
Xu et al., 2012b	YanRenMing Standard	RCT	GDL+DMPS	DMPS	41	29/12a 17.64 ± 6.28 5.56 ± 4.55y		64 d	1. The indicator of portal circulation PVFV,SVFV2. 24 h excretion of urinary copper3. Adverse effect	1. *p* < 0.05 2. *p* < 0.01
Wang et al., 2010	YanRenMing Standard	RCT	GDL+DMPS	DMPS	112	32/26 21.6 ± 9.17 3 mo to 31 y	36/18 2.16 ± 10.79 5 mo to 29 y	6 mo	1. Clinical symptoms 2. T cell,CD3+,CD4+, CD8+, 3. NK cell	1. *p* > 0. 052. *p* > 0. 053. *p* < 0. 05
Chen and Wang, 2010	YanRenMing Standard	RCT	CHGD	Penicillamine	88	32/27; 21.24 ± 11.32; 6—34 mo	16/13; 20.98 ± 10.75; 7−33 mo	90 d	1.Clinical symptoms 2.24 h excretion of urinary copper 3.Blood Cu2+ and CP 4.Liver function 5.Adverse effect	1.*p* > 0. 052. *p* < 0.053. *p* > 0.054. *p* < 0.05
Chen and Wang, 2008	Sternlieb standard	RCT	SGLDPD	Penicillamine	61	29/11; 21.12 ± 10.96; 8−35 mo	14/7; 20.81 ± 10.46; 8−34 mo	90 d	1. Hepaticul trasonography 2. Liver function 3. 24 h excretion of urinary copper 4. Cornea Kayser—Fleischer rings 5. Adverse effect	1. *p* < 0. 052. *p* < 0.053. *p* < 0.014. *p* > 0.05
Zhang, 2007	YanRenMing Standard	RCT	DHGD + Penicillamine	Penicillamine	40	12/8; – 3mo−2.18y	11/9;- 5 mo to 2.25 y	30 d	1.Clinical symptoms 2. 24 h excretion of urinary copper	1. *p* < 0.052. *p* < 0.05
Xue et al., 2007	Yang RenMin Standard	RCT	GDT No. 2 +DMPS	DMPS	61	17/14 23.1 ± 7.8 9 mo-3.5 y	17/13 22.3 ± 8.5 6 mo to 4 y	62 d	1.Liver function 2.The indicator of hepatic fibrosis	1. *p* < 0.052. *p* > 0.05,
Xiao, 2003	Homemade standard	RCT	RJ+penicillamine and Zincsulfate	Penicillamine and Zinc sulfate	38	22/16a 5-−13 y		3 mo	1.Clinical symptoms2.Liver function3.Index of hepatic fibrosis	1. *p* < 0.05 2.*p* < 0.05 3.*p* < 0.05
Cui and Zhao, 2001	Shi Yuquan standard	RCT	GDT +Zincsulfate	Zincsulfate	33	10/7 19.8 ± 2.93y 4 mo to 6 y	11/5 20.14 ± 2.6y 6 mo to 7y	4 w	1. Clinical symptoms 2. 24 hexcretion ofurinary copper	1. *p* > 0. 052. *p* < 0.05
Hong et al., 2000	Yang RenMing Standard	RCT	GDP +DMPS	DMPS	146	31/19 18.6 ± 2.7y 2.6 ± 0.8y	26/24 18.9 ± 6.8y 3.1 ± 1.2y	8 w	1.Hepatic ultrasonography 2.Electrophoresis of serumprotein 3.24 hexcretion ofurinary copper	1. *p* < 0. 012. *p* > 0. 053. *p* < 0. 05
Han et al., 1999	Yang RenMing Standard	RCT	GDP	DMSA	94	21/11; 17.6 ± 7.2; 3 mo to 7 y	38/24; 19.0 ± 4.1; 2 mo to 14 y	4 w	1. Clinical symptoms 2. 24hexcretion ofurinary copper 3. Adverse effect	1. *p* > 0. 052. *p* < 0.01
Ren et al., 1997	Shi Yuquan standard	RCT	GDT+DMSA	DMSA	80	21/19; 20.48 ± 10.90; 4 mo to 6 y	22/18; 19.65 ± 7.18; 6 mo to 5 y	4 w	1. Clinical symptoms 2. 24hexcretion ofurinary copper 3. Adverse effect	1. *p* < 0. 052. *p* > 0. 05

*GDL, Gandouling Tablet; SGLDPD, Shugan Lidan Paidu Decoction; CHGD, Chaihuang Gandou Powder; GDT, Gandou Tang; GDP, Gandou Pian; RJ, Ruanjian Syrup; GDT No. 2, Gandou Tang No. 2; DHGD, Dahuang Gandou Decoction; CMAN, Chinese medical association of neurology; CP, copper-protein; d, day; DMPS, sodium dimercaptopropanesulfonate; DMSA, dimercaptosuccinate acid; EDTA, calcium disodium ethylene diaminotetraacetate; EF, ejection fraction; FEV1,forcedexpiratory volume at 1 sec; FVC, forced vital capacity; h, hour; mo, month; MMSE, Mini-mental State Examination; MoCA, Montreal Cognitive Assessment; PVF, portal venous flow; RCT, randomized controlled trial; SVF, splenic vein flow; TECT, tiopronin enteric-coated tablet; w, week*.

**Table 2:** Ingredients, usage and quality control of CHM.

**References**	**Prescription name**	**Ingredients of herb prescription**	**Usage of prescription**	**Preparations**	**Quality control**
Jiang, 2016	GDL	Radix Curcumae, Radix Salviae Miltiorrhizae, Caulis Spatholobi, Rhizoma Acori Tatarinowii, Rhizoma Curcumae Longae, Rhizoma Curcumae, Rhizoma Coptidis, Radix et Rhizoma Rhei, Herba Scutellariae Barbatae, Herba Andrographis	5#tid	Tablet	Traditional Chinese patented medicine WY:Z20050071
Fang, 2015	GDL	Radix Curcumae, Radix Salviae Miltiorrhizae, Caulis Spatholobi, Rhizoma Acori Tatarinowii, Rhizoma Curcumae Longae, Rhizoma Curcumae, Rhizoma Coptidis, Radix et Rhizoma Rhei, Herba Scutellariae Barbatae, Herba Andrographis	5# tid	Tablet	Traditional Chinese patented medicine WY:Z20050071
Zhang et al., 2014a	GDL	Radix Curcumae, Radix Salviae Miltiorrhizae, Caulis Spatholobi, Rhizoma Acori Tatarinowii, Rhizoma Curcumae Longae, Rhizoma Curcumae, Rhizoma Coptidis, Radix et Rhizoma Rhei, Herba Scutellariae Barbatae, Herba Andrographis	5#tid	Tablet	Traditional Chinese patented medicine WY:Z20050071
Zhang et al., 2014b	GDT	Radix et Rhizoma Rhei, Rhizoma Coptidis, Radix Scutellariae, Herba Andrographis, Herba Scutellariae Barbatae, Rhizoma Dioscoreae Hypoglaucae, Cortex Phellodendri, Rhizoma Alismatis, Herba Houttuyniae	200 mL qd po	Decoction	UR
Hu, 2014	GDT	Radix et Rhizoma Rhei, Rhizoma Coptidis, Radix Scutellariae, Herba Andrographis, Herba Scutellariae Barbatae, Rhizoma Dioscoreae Hypoglaucae, Cortex Phellodendri, Rhizoma Alismatis, Herba Houttuyniae	1# bid po	Decoction	UR
Han et al., 2014	GDL	Radix Curcumae, Radix Salviae Miltiorrhizae, Caulis Spatholobi, Rhizoma Acori Tatarinowii, Rhizoma Curcumae Longae, Rhizoma Curcumae, Rhizoma Coptidis, Radix et Rhizoma Rhei, Herba Scutellariae Barbatae, Herba Andrographis	3–5g (80 mg/kg) tid po	Tablet	Traditional Chinese patented medicine WY:Z20050071
Xu et al., 2012a	GDT	Radix et Rhizoma Rhei, Rhizoma Coptidis, Radix Scutellariae, Herba Andrographis, Herba Scutellariae Barbatae, Rhizoma Dioscoreae Hypoglaucae, Cortex Phellodendri, Rhizoma Alismatis, Herba Houttuyniae	1# bid po	Decoction	UR
Xu et al., 2012b	GDL	Radix Curcumae, Radix Salviae Miltiorrhizae, Caulis Spatholobi, Rhizoma Acori Tatarinowii, Rhizoma Curcumae Longae, Rhizoma Curcumae, Rhizoma Coptidis, Radix et Rhizoma Rhei, Herba Scutellariae Barbatae, Herba Andrographis	UR	Tablet	Traditional Chinese patented medicine WY:Z20050071
Wang et al., 2010	GDL	Radix Curcumae, Radix Salviae Miltiorrhizae, Caulis Spatholobi, Rhizoma Acori Tatarinowii, Rhizoma Curcumae Longae, Rhizoma Curcumae, Rhizoma Coptidis, Radix et Rhizoma Rhei, Herba Scutellariae Barbatae, Herba Andrographis	5#tid	Tablet	Traditional Chinese patented medicine WY:Z20050071
Chen and Wang, 2010	CHGD	Radix Bupleuri, Radix et Rhizoma Rhei, Herba Lysimachiae, Herba Artemisiae Scopariae, Radix Aucklandiae, Pericarpium Citri Reticulatae Viride, Rhizoma Alismatis, Rhizoma Dioscoreae Hypoglaucae, Caulis Spatholobi, Radix Salviae Miltiorrhizae	5 g tid po	powder	Hospital Preparation
Chen and Wang, 2008	SGLDPD	Herba Lysimachiae 30 g, Radix Bupleuri 15 g, Radix Curcumae 15 g, Herba Artemisiae Scopariae 15 g, Rhizoma Alismatis15 g, Pericarpium Citri Reticulatae Viride 20 g, Pericarpium Citri Reticulatae 20 g, Rhizoma Dioscoreae Hypoglaucae 12 g, Radix Clematidis 18 g, Caulis Spatholobi 18 g, Rhizoma Ligustici Chuanxiong 9 g, Radix et Rhizoma Rhei 9 g	196 g qd po	Decoction	UR
Zhang, 2007	DHGD	Rhizoma Polygonati 20 g, Radix et Rhizoma Rhei 10 g, Herba Lysimachiae 20 g, Gypsum Fibrosum 9 g, Radix Curcumae 9 g, Radix Angelicae Sinensis 20 g, Radix Salviae Miltiorrhizae 15 g, Radix Asparagi 15 g, Poria 20 g, Flos Chrysanthemi 9 g, Radix Paeoniae Alba 15 g, Pericarpium Citri Reticulatae 9 g, Rhizoma Atractylodis 9 g, Rhizoma Acori Tatarinowii 6 g	250 ml# bid po	Decoction	UR
Xue et al., 2007	GDT No. 2	Radix et Rhizoma Rhei, Radix Salviae Miltiorrhizae, Radix Sophorae Flavescenti, Radix Astragali seu Hedysari, Rhizoma Alismatis	1# qd po	Decoction	UR
Xiao, 2003	RJ	Radix Codonopsis, Radix Bupleuri, Radix Paeoniae Rubra, Radix Paeoniae Alba, Rhizoma Sparganii, Rhizoma Curcumae, Radix Curcumae, Concha Ostreae, Fructus Lycii	15–30 ml tid po	syrup	Hospital Preparation
Cui and Zhao, 2001	GDT	Radix et Rhizoma Rhei 6–9 g, Rhizoma Coptidis 20 g, Radix Scutellariae 20 g, Herba Scutellariae Barbatae 20 g, Herba Andrographis 20 g, Rhizoma Dioscoreae Hypoglaucae 20 g	250 ml bid po	Decoction	UR
Hong et al., 2000	GDP	Radix et Rhizoma Rhei 0.25 g, Rhizoma Coptidis 0.25 g, Rhizoma Curcumae Longae 0.25 g, Herba Lysimachiae 0.625 g, Rhizoma Alismatis 0.625 g, Radix Notoginseng 0.042 g	< 15 years old: 6# tid po ≥15 years old: 8# tid po	Tablet	Hefei Chinese Medicine Factory
Han et al., 1999	GDP	Radix et Rhizoma Rhei 0.25 g, Rhizoma Coptidis 0.25 g, Rhizoma Curcumae Longae 0.25 g, Herba Lysimachiae 0.625 g, Rhizoma Alismatis 0.625 g, Radix Notoginseng 0.042 g	< 15 years old: 6# tid po ≥15 years old: 8# tid po	Tablet	Hefei Chinese Medicine Factory
Ren et al., 1997	GDT	Radix et Rhizoma Rhei, Rhizoma Coptidis, Radix Scutellariae, Herba Andrographis, Herba Scutellariae Barbatae, Rhizoma Dioscoreae Hypoglaucae, Cortex Phellodendri, Rhizoma Alismatis, Herba Houttuyniae	1# bid po	Decoction	UR

*GDL, Gandouling Tablet; SGLDPD, Shugan Lidan Paidu Decoction; CHGD, Chaihuang Gandou Powder; GDT, Gandou Tang; GDP, Gandou Pian; RJ, Ruanjian Syrup; GDT No. 2, Gandou Tang No. 2; DHGD, Dahuang Gandou Decoction; UR, Unreported.bid, bis in die; d:day; po, peros; qd, quaquedie; tid, ter in die;#, tablet*.

### The Reporting Completeness of the Clinical Studies

We accessed the reporting completeness of the material in each study with a rating system, which is related to the information about the botanical material and voucher specimens. Only two studies (Han et al., 2014; Zhang et al., 2014a) are of high quality, which provided the full information about the botanical material and included voucher specimens. Twelve studies (Ren et al., 1997; Han et al., 1999; Hong et al., 2000; Cui and Zhao, 2001; Xiao, 2003; Xue et al., 2007; Zhang, 2007; Chen and Wang, 2008, 2010; Xu et al., 2012a; Hu, 2014; Zhang et al., 2014b) are of moderate quality, which provided partial information about the botanical material and did not provide voucher specimens. Four studies (Wang et al., 2010; Xu et al., 2012b; Fang, 2015; Jiang, 2016) are of low quality with inadequate information and were overall taxonomically inadequate. The quality of each included clinical study is summarized in Table 3.

**Table 3:** The quality of the clinical studies.

**References**	**Botanical material information**	**Voucher specimen**	**Quality**
Zhang et al., 2014a	P	+	High
Han et al., 2014	P	+	High
Zhang et al., 2014b	P	–	Moderate
Hu, 2014	P	–	Moderate
Xu et al., 2012a	P	–	Moderate
Chen and Wang, 2010	P	–	Moderate
Chen and Wang, 2008	P	–	Moderate
Zhang, 2007	P	–	Moderate
Xue et al., 2007	P	–	Moderate
Xiao, 2003	P	–	Moderate
Cui and Zhao, 2001	P	–	Moderate
Hong et al., 2000	P	–	Moderate
Han et al., 1999	P	–	Moderate
Ren et al., 1997	P	–	Moderate
Jiang, 2016	I	+	Low
Fang, 2015	I	+	Low
Xu et al., 2012b	I	+	Low
Wang et al., 2010	I	+	Low

*F, Full information about the botanical material is provided; P, Partial information about the botanical material is provided; I, Inadequate information about the botanical material is provided; +, includes a voucher specimen; –, a voucher specimen is missing*.

### Risk of Bias in Included Studies

The score of RoB ranged from 2/7 to 4/7. Of which, 10 studies got two points (Ren et al., 1997; Cui and Zhao, 2001; Xiao, 2003; Xue et al., 2007; Zhang, 2007; Chen and Wang, 2008, 2010; Xu et al., 2012b; Fang, 2015; Jiang, 2016); 7 studies got three points (Han et al., 1999; Hong et al., 2000; Wang et al., 2010; Xu et al., 2012a; Hu, 2014; Zhang et al., 2014a,b); and 1 study got four points (Han et al., 2014). Two studies (Xu et al., 2012a; Han et al., 2014) described the detailed methods for random sequence generation and no studies described allocation concealment. No blinding on patients or personnel was applied. All studies reported drop-out data. Ten studies (Cui and Zhao, 2001; Xiao, 2003; Xue et al., 2007; Zhang, 2007; Chen and Wang, 2008, 2010; Xu et al., 2012a,b; Fang, 2015; Jiang, 2016) were judged as unclear risk of bias for selective reporting. There were baseline comparisons and patients' consent were well reported, and other biases were not found in all included studies. The RoB in each included study is concluded in Table 4.

**Table 4:** Risk of bias of the included studies.

**Included studies**	**A**	**B**	**C**	**D**	**E**	**F**	**G**	**Total**
Jiang, 2016	?	0	0	0	1	?	1	2
Filippi and Dhawan, 2014	?	0	0	0	1	?	1	2
Zhang et al., 2014a	?	0	0	0	1	1	1	3
Zhang et al., 2014b	?	0	0	0	1	1	1	3
Hu, 2014	?	0	0	0	1	1	1	3
Han et al., 2014	1	0	0	0	1	1	1	4
Xu et al., 2012a	1	0	0	0	1	?	1	3
Xu et al., 2012b	?	0	0	0	1	?	1	2
Wang et al., 2010	?	0	0	0	1	1	1	3
Chen and Wang, 2010	?	0	0	0	1	?	1	2
Chen and Wang, 2008	?	0	0	0	1	?	1	2
Zhang, 2007	?	0	0	0	1	?	1	2
Xue et al., 2007	?	0	0	0	1	?	1	2
Xiao, 2003	?	0	0	0	1	?	1	2
Cui and Zhao, 2001	?	0	0	0	1	?	1	2
Hong et al., 2000	?	0	0	0	1	1	1	3
Han et al., 1999	?	0	0	0	1	1	1	3
Ren et al., 1997	?	0	0	0	1	1	1	2

*Cochrane Collaboration's tool: A, Random sequence generation; B, Allocation concealment; C, Blinding of participants or personnel; D, Blinding of outcome assessment; E, Incomplete outcome data; F, Selective reporting; G, Anything else; 1, Low risk of bias; 0, High risk of bias; ?, Uncertain risk of bias*.

### Effectiveness

#### CHM vs. Placebo

None of RCTs used a specific comparison between CHM and placebo.

#### CHM vs. WCM

**Figure 2:**
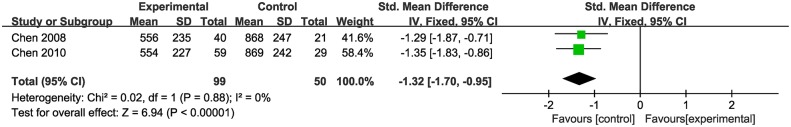
The forest plot: The 24 h excretion of urinary copper of CHM vs. WCM.

Two studies (Chen and Wang, 2008, 2010) showed that CHM monotherapy had no significance for increasing the amount of copper excreted in the urine in a 24 h period (*n* = 149, SMD−1.32, 95% CI [−1.70 to −0.95], *p* < 0.01; heterogeneity: χ^2^ = 0.02, df = 1 (*p* = 0.88); *I*^2^ = 0%) (Figure 2).

#### CHM Plus WCM vs. WCM

##### 24 h excretion of urinary copper

**Figure 3:**
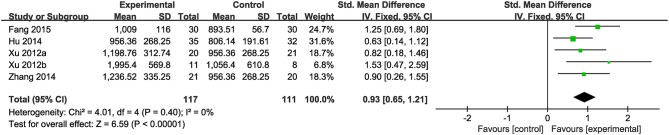
The forest plot: The 24 h excretion of urinary copper of CHM plus WCM vs. WCM.

Nine studies were included. Meta-analysis of 5 studies (Xu et al., 2012a,b; Hu, 2014; Zhang et al., 2014b; Fang, 2015) reported a significant effect of CHM on increasing the amount of 24 h excretion of urinary copper in patients with WD compared to the control (*n* = 228, SMD 0.93, 95% CI [0.65 to 1.21], *p* < 0.01;heterogeneity: χ^2^ = 4.01, df = 4 (*p* = 0.40); *I*^2^ = 0%) (Figure 3). Four studies (Ren et al., 1997; Hong et al., 2000; Cui and Zhao, 2001; Zhang, 2007) failed for pool analysis because the measurement unit of 24 h excretion of urinary copper was different from the remaining. However, they all got the significant effects of improving the 24 h excretion of urinary copper on patients (*p* < 0.05).

##### Liver function and the indicator of hepatic fibrosis

**Figure 4:**
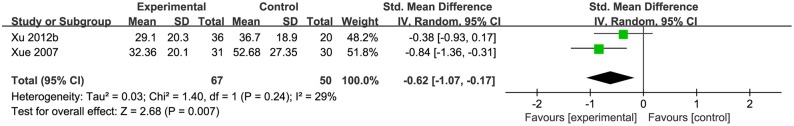
The forest plot: The liver function of CHM plus WCM vs. WCM.

Two studies (Xue et al., 2007; Xu et al., 2012a) used the value of serum alanine aminotransferase (ALT) as the indicator of liver function. Pooled data showed that CHM was significantly better at decreasing the ALT compared with control group [*n* = 117, SMD−0.62, 95% CI [−1.00 to −0.24], *p* < 0.01; heterogeneity: χ^2^ = 1.40, df = 1 (*p* = 0.24); *I*^2^ = 29%], (Figure 4). One study (Xiao, 2003) used ALT recovery rate as the indicator of liver function, and it demonstrated significant effects on decreasing the ALT (*p* < 0.05). One study (Xiao, 2003) showed that CHM had significant effects on reducing HA, PCIII, and LN (*p* < 0.05), however, another study (Xue et al., 2007) showed that CHM had no effect on reducing HA, PCIII and LN in short time (*p* > 0.05).

##### The total clinical effective rate

**Figure 5:**
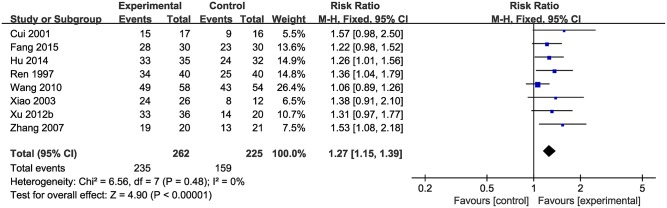
The forest plot: the total clinical effective rate of CHM plus WCM vs. WCM.

Data on the rate of total clinical effectiveness were available from eight studies with 487 participants included. Meta-analysis of 8 studies showed a significant effect of CHM on increasing the total clinical effective rate compared with control group (*n* = 487, RR 1.27, 95% CI [1.15 to 1.39], *p* < 0.01; heterogeneity: χ^2^ = 6.56, df = 7 (*p* = 0.48), *I*^2^ = 0%) (Figure 5).

##### Laboratory values or imaging indices

One study (Xu et al., 2012b) showed that CHM paratherapy is significant for increasing portal venous flow (PVF) and splenic vein flow (SVF) (*p* < 0.05) compared with WCM, whereas another study (Zhang et al., 2014b) showed no difference. One study (Fang, 2015) showed that CHM could significantly improve the cardiac function according to electrocardiogram, ejection fraction, and myocardial enzyme spectrum relative to WCM (*p* < 0.05). One study (Han et al., 2014) showed that CHM is significant for improving the Mini-mental State Examination (MMSE) and Montreal Cognitive Assessment (MoCA) (*p* < 0.05) compared with WCM.

#### Adverse Events

Adverse effects were reported in 8 studies (Ren et al., 1997; Han et al., 1999; Chen and Wang, 2008, 2010; Xu et al., 2012a,b; Hu, 2014; Fang, 2015). There were no significant differences in routine blood, routine urine, routine stool, and osteoporosis after CHM treatment in three studies (Ren et al., 1997; Xu et al., 2012a,b). Five studies (Han et al., 1999; Chen and Wang, 2008, 2010; Hu, 2014; Fang, 2015) reported that CHM could significantly reduce the adverse events of acne, gastrointestinal reaction, joint pain, and blood reduction compared with WCM. However, life-threatening adverse effects were not mentioned in all of these studies.

### Description of the CHM

Twenty-five herbs were included in the 18 studies. The top 13 most frequently used herbs were *Radix et Rhizoma Rhei, Rhizoma Coptidis, Rhizoma Curcumae Longae, Rhizoma Curcumae, Radix Salviae Miltiorrhizae, Herba Andrographis, Herba Lysimachiae, Herba Scutellariae Barbatae, Caulis Spatholobi, Rhizoma Alismatis, Radix Curcumae, Radix Scutellariae* and *Rhizoma Acori Tatarinowii*, and all of them were used more than 5 times. The full and validated botanical names of herbs were generalized in Table 5.

**Table 5:** Details of the most commonly used herbs for WD.

**Chinese name**	**Pharmaceutical name**	**Species**	**Family**	**Record**	**N/18 (%)**
Dahuang	*Radix et Rhizoma Rhei*	*Rheum officinale* Baill.	*Polygonaceae*	–	17(94%)
Huanglian	*Rhizoma Coptidis*	*Coptis chinensis* Franch.	*Ranunculaceae*	–	13(72%)
Banzhilian	*Herba Scutellariae Barbatae*	*Scutellaria barbata* D.Don	*Lamiaceae*	188943	11(61%)
Chuanxinlian	*Herba Andrographis*	*Andrographis paniculata* (Burm.f.) Nees	*Acanthaceae*	–	11(61%)
Danshen	*Radix Salviae Miltiorrhizae*	*Salvia miltiorrhiza* Bunge	*Lamiaceae*	183206	9(50%)
Zexie	*Rhizoma Alismatis*	*Alisma orientale* (Sam.) Juz.	*Alismataceae*	294832	9(50%)
Ezhu	*Rhizoma Curcumae*	*Curcuma phaeocaulis* Valeton	*Zingiberaceae*	235270	9(50%)
Yujin	*Radix Curcumae*	*Curcuma wenyujin* Y.H.Chen & C.Ling	*Zingiberaceae*	235308	9(50%)
Jianghuang	*Rhizoma Curcumae Longae*	*Curcuma longa* L.	*Zingiberaceae*	235249	8(44%)
Shichangpu	*Rhizoma Acori Tatarinowii*	*Acorus tatarinowii* Schott	*Acoraceae*	2337	7(39%)
Jixueteng	*Caulis Spatholobi*	*Spatholobus suberectus* Dunn	*Leguminosae*	32974	8(44%)
Huangqin	*Radix Scutellariae*	*Scutellaria baicalensis* Georgi	*Lamiaceae*	188938	5(28%)
Jinqiancao	*Herba Lysimachiae*	*Lysimachia christinae* Hance	*Primulaceae*	–	5(28%)

### The Possible Mechanisms of CHM for WD

The possible mechanisms of the most frequently used herbs and the main active ingredients are as follows: (1) *Curcumin*: an active ingredient from commonly used herbs like *Rhizoma Curcumae Longae, Rhizoma Curcumae, Radix Curcumae* and *Radix Curcumae* can partially restore protein expression of most ATP7B mutants to restore functional copper export (van den Berghe et al., 2000; Zhang et al., 2011; European Association for Study of Liver, 2012). Furthermore, curcumin is an ideal antioxidant, an effective scavenger of reactive oxygen species (Samarghandian et al., 2017), and it exerts anti-fibrotic effect through regulating hepatic stellate cells (HSCs) function (Jin et al., 2016; Liu et al., 2016; Mustafa, 2016). (2) *Radix et Rhizoma Rhei*: Rhubarb root and its active components have anti-oxidation (Shia et al., 2009), anti-fibrotic (Jin et al., 2005), and anti-inflammation effects (Hwang et al., 2013). (3) *Rhizoma Coptidis*: Berberine from *Rhizoma Coptidis* exerted anti-fibrotic and anti-oxidation effects (Zhang et al., 2008). (4) *Herba Scutellariae Barbatae*: P-coumaric acid from *Herba Scutellariae Barbatae* possess anti-oxidative activities (Ibrahim et al., 2007) and reverse the ATP7B function defect via regulating pre-mRNA splicing (Lin et al., 2015). (5) *Herba Andrographis*: Andrographolide from *Herba Andrographis* displayed anti-inflammatory activity through reducing the expression of pro-inflammatory mediators (Panossian et al., 2002) and exhibited hepatoprotective effects through anti-oxidative effect (Vetriselvan et al., 2011).

## Discussion

### Summary of Evidence

Eighteen RCTs involving 1,220 patients suffering from WD were identified. The main findings of this study were that CHM adjuvant therapy could increase 24 h urinary copper excretion, and improve liver function and the total clinical efficacy rate for WD. Two trails (Chen and Wang, 2008, 2010) indicated that CHM monotherapy was not superior to the WCM. Eight out of eighteen studies reported no serious adverse events relevant to CHM formulas, indicating that CHM formulas were generally safe and well tolerated for patients with WD. The possible mechanisms are associated with reversing the ATP7B mutants, and exerting anti-oxidation, anti-inflammation and anti-fibrotic effects. Thus, the findings of the present study suggested, to a limited extent, that CHM paratherapy can be used for WD according to the methodological flaws, whereas the beneficial use of CHM monotherapy for WD still lacks evidence.

### Limitations

There are several limitations in the primary studies. Firstly, although we included RCTs, some inherent and methodological weaknesses still existed in the primary studies: only 2 trials provided sufficient information on how the random allocation was generated, while none of the other trials included reported the allocation concealment. No study employed the blinding procedure, making it difficult to bias results intentionally or unintentionally and to help ensure the credibility of study conclusions. A placebo effect is conceptually defined as the beneficial effect associated with an intervention that does not include the presumed active ingredients; however, CHM placebo are hard to mimic identical interventional herbal prescription due to the fact that CHM is special in color, smell and taste. Thus, placebo-controlled randomized trials are well-recognized method when evaluating the efficacy of CHM treatment. In addition, most trials are without calculating the formal pretrial sample size. The trials with inadequate sample sizes seem to be one risk in exaggerating intervention benefits. Secondly, WD is a chronic disease, which needs life-long treatments. Long-term efficacy and safety are important assessments to determine the clinical effectiveness of an agent in treatment. However, in the present study, treatment duration ranged from 28 to 90 days. Long-term safety of CHM for WD could not be determined because duration of treatment is short and dropouts were only reported in one study. According to other clinical trials for WD (Brewer et al., 2009; Weiss et al., 2015; Nicholl et al., 2017), it is recommended that the treatment duration of further trials must not be >60 days, and must last more than 1 year. Thirdly, clinical heterogeneity would be very significant due to the variations in study quality, intervention of CHM prescriptions, comparators, and outcome measures. Owing to being highly variable in composition and dosage of CHMs, it is difficult to assess the efficacy of a specific CHM by performing pooling analysis. Fourthly, all trials were conducted in China, which may limit the generalizability. Further international multicenter RCTs of CHM for WD are needed, in order to generalize the results worldwide.

### Implications for Practice

Use of CHM for WD patients has increased in the past decades. However, the choice of CHM is mainly empirical and lacking consensus among clinical doctors. The available evidence from the present study supported, to a limited extent, that CHM paratherapy can be used for patients with WD but should not be recommended as monotherapy in WD. In addition, the most frequently used herbs selected by the present study should be considered as herbal prescription for WD and as a candidate for further clinical trials.

### Implications for Research

In the present study, we identified an area that is worthy of further study. Firstly, the potential benefit of CHM as an adjunct treatment for WD still needs to be further confirmed by high-quality RCTs. Thus, we recommend that CONSORT 2010 statement (Schulz et al., 2010), CONSORT for CHM Formulas (Cheng et al., 2017), and RCTs investigating CHM (Flower et al., 2011) should be used as the guidelines when the designing, registering and reporting of further RCTs. Secondly, WD was thought of as a “rare” autosomal disorder by neurologists, and it proved difficult to conduct large sample RCT. However, this review identified 1,220 subjects with WD from 1997 to 2016. If the primary clinical data of all RCTs were recorded in standard, the evidence would be more reliable. Thus, it is necessary to promote clinical data sharing, as has been suggested by the International Committee of Medical Journal Editors (ICMJE) (Taichman et al., 2017).

WD is caused by ATP7B mutations, resulting in copper accumulation and toxicity. The possible mechanisms of CHM for WD not only involve the targets of the ATP7B gene, but also the multiple targets of copper accumulation in various tissues and organs. Curcumin and P-coumaric acid were reported to reverse the ATP7B function defect. Curcumin could partially restore protein expression by directly enhancing the protein expression of mutant ATP7B with residual copper export activity (van den Berghe et al., 2000; Zhang et al., 2011; European Association for Study of Liver, 2012). The EASL Guidelines recommended that treatment with curcumin might be a novel therapeutic strategy in WD (European Association for Study of Liver, 2012). P-coumaric acid, another ingredient of herbs, can also reverse the ATP7B function defect via a different mechanism by regulating pre-mRNA splicing (Lin et al., 2015).

**Figure 6:**
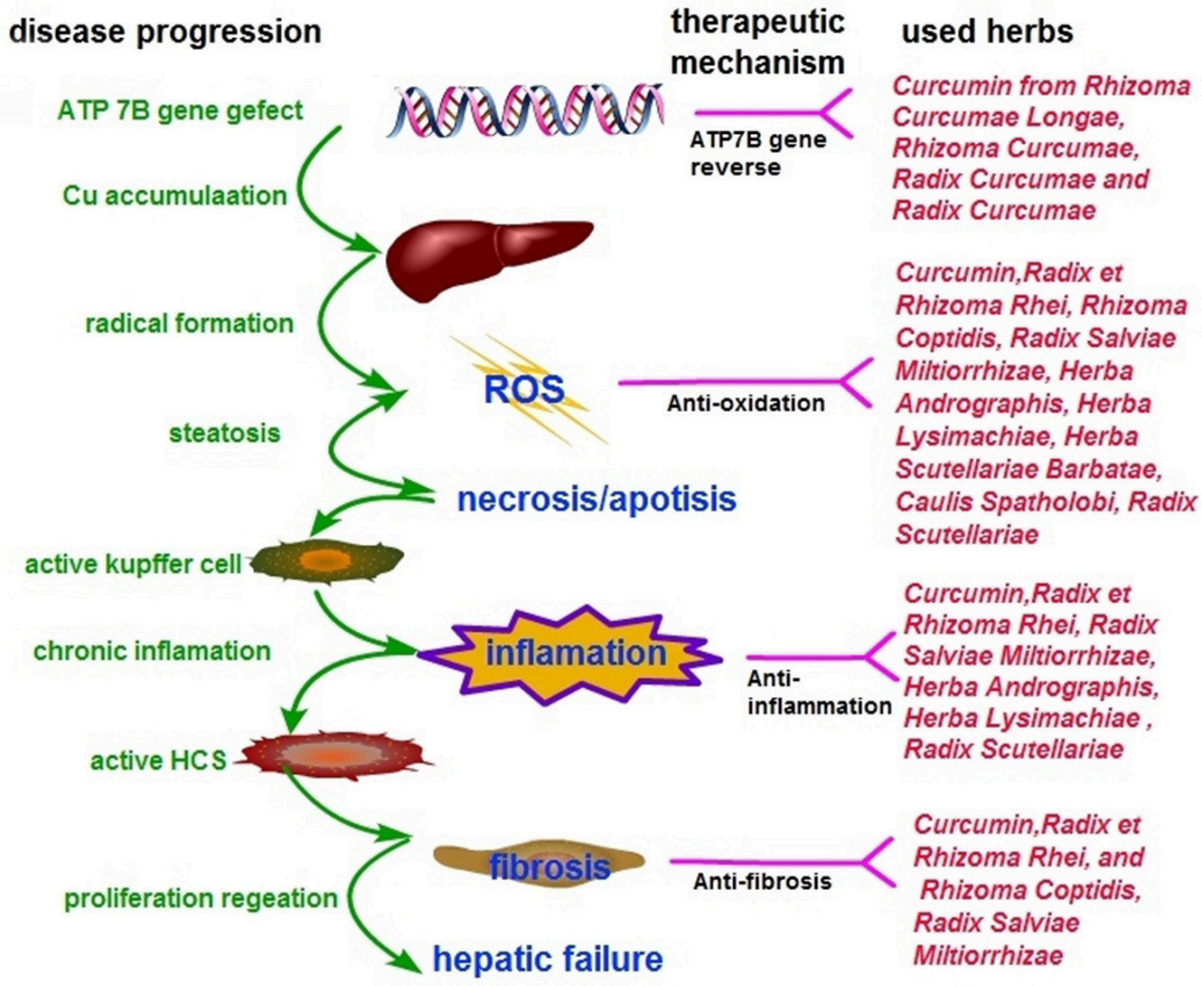
Simplified model of copper toxicity and disease progression and potential targets for CHM intervention.

Copper accumulates in hepatocytes where it induces damage through oxidative stress due to its highly reactive redox capacity (Rosencrantz and Schilsky, 2011). In addition, necrosis and apoptosis triggered immune reaction and inflammation to activate the quiescent HSCs, causing hepatic fibrosis (Jin et al., 2016). The possible pharmacological mechanisms of CHM for copper accumulations of WD are as follows: (1) Antioxidant effects: Curcumin, Anthraquinone (from *Radix et Rhizoma Rhei*), Danshensu and Salvianolic acid B (from *Radix Salviae Miltiorrhizae*), were shown to ameliorate the oxidative stress by reducing oxidative stress parameters malondialdehyde, thereby improving the hepatic glutathione content and hepatic superoxide dismutase (SOD) (Liu et al., 2016; Samarghandian et al., 2017), inhibiting the formation of superoxide anions (Shia et al., 2009), and exerting a low level of lipid peroxidase (Mishra et al., 2014; Lee et al., 2016, 2017), leading to maintenance of mitochondrial activity (Zhou et al., 2015). *Radix Scutellariae* improved the antioxidant capacity by induction of the antioxidative enzymes and removal of reactive oxygen species (ROS) (Pan et al., 2015). P-coumaric acid (Ibrahim et al., 2007), Andrographolideand (Vetriselvan et al., 2011), *Caulis Spatholobi* (Jeon et al., 2008) and Tanshinone IIA, (Shu et al., 2016) have also been shown to exhibit antioxidant effects; (2) Anti-inflammatory effects: Emodin (from *Radix et Rhizoma Rhei*), Andrographolide (from *Herba Andrographis*), *Radix Salviae Miltiorrhizae* and Curcumin analog demonstrated anti-inflammatory properties by reducing the expression of pro-inflammatory mediators via the NF-kB activation pathway (Lee et al., 2003; Hwang et al., 2013; Yue et al., 2014) and MAPK/AP-1 pathway (Choi et al., 2013), and by inhibiting iNOS and COX-2 expression (Paulino et al., 2016). The bioactive components from *Radix Scutellariae* (Liu et al., 2016) and Quercetin from *Herba Lysimachiae* (Wang et al., 2015) have been reported to exhibit anti-inflammatory activity; (3) Anti-fibrotic effects: activation of quiescent HSCs is the major event in hepatic fibrosis (Jin et al., 2016). Skullcapflavone I (from *Radix Scutellariae*) (Park et al., 2005) and Curcumin (Jin et al., 2016) exerted anti-fibrotic effects by inducing apoptosis or senescence in activated HSCs. Furthermore, Curcumin was found to be an anti-fibrotic mediator that inhibits HSCs activation and the transition to myofibroblast-like cells (Mustafa, 2016). In contrast, *Radix et Rhizoma Rhei* exerted anti-fibrotic effects by the direct inhibition of stellate cell activation without reducing hepatocyte cell death (Jin et al., 2005). Salvianolic acid A and B from Danshen (Tsai et al., 2011), Berberine (Zhang et al., 2008), *Radix Scutellariae* (Chen et al., 2013) and *Radix et Rhizoma Rhei* (Pan et al., 2015) have been reported to prevent hepatic fibrosis in different aspects, including inhibition of proliferation and fibrogenesis of HSCs, and regulation of the antioxidant system and lipid peroxidation. Thus, CHM is likely to be useful as a multi-targeting therapy for WD pathogenesis (Figure 6).

## Conclusion

Despite the apparent positive results, the present evidence supports, to a limited extent because of the methodological flaws and CHM heterogeneity, that CHM paratherapy can be used for patients with WD but should not be recommended as monotherapy in WD. The possible mechanisms involved are associated with reversing the ATP7B mutants, and exerting anti-oxidation, anti-inflammation and anti-hepatic fibrosis effects. Further rigorous RCTs, focusing on an individual CHM formula for WD, are warranted.

## Author Contributions

G-QZ contribute as the senior authors and the principal investigator (PI) of this study. M-BX, P-QR, and T-YJ wrote the first draft of the manuscript and contributed to the overall design. G-QZ refined the study. P-PZ and H-YL identified reviewed studies for eligibility and performed the meta-analysis of data. All authors read, critically reviewed and approved the final manuscript.

### Conflict of Interest Statement

The authors declare that the research was conducted in the absence of any commercial or financial relationships that could be construed as a potential conflict of interest.
